# Decomposition Analysis of the Carbon Footprint of Primary Metals

**DOI:** 10.1021/acs.est.2c05857

**Published:** 2023-05-05

**Authors:** Kajwan Rasul, Edgar G. Hertwich

**Affiliations:** Industrial Ecology Programme, Department of Energy and Process Engineering, Norwegian University of Science and Technology, Høgskoleringen 1, Trondheim 7034, Norway

**Keywords:** iron and steel, aluminum, copper, nonferrous metals, energy intensity of metal production, environmental Kuznets curve (EKC), mineral resources, global supply chain

## Abstract

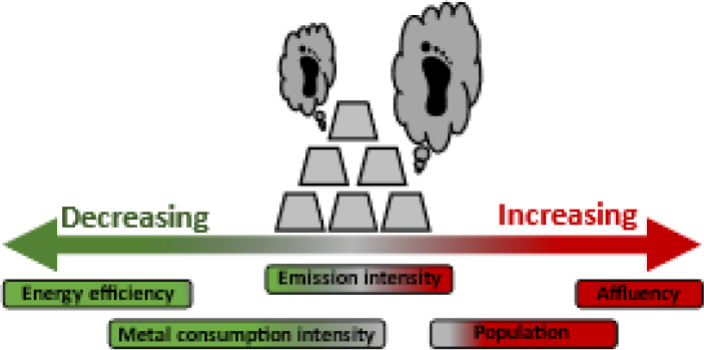

This study investigates how different technological and socioeconomic drivers have impacted the carbon footprint of primary metals. It analyzes the historical evidence from 1995 to 2018 using new metal production, energy use, and greenhouse gas (GHG) emission extensions made for the multiregional input–output model EXIOBASE. A combination of established input–output methods (index decomposition analysis, hypothetical extraction method, and footprint analysis) is used to dissect the drivers of the change in the upstream emissions occurring due to the production of metals demanded by other (downstream) economic activities. On a global level, GHG emissions from metal production have increased at a similar pace as the GDP but have decreased in high-income countries in the most recent 6 year period studied. This absolute decoupling in industrialized countries is mainly driven by reduced metal consumption intensity and improved energy efficiency. However, in emerging economies increasing metal consumption intensity and affluency have driven up emissions, more than offsetting any reductions due to improved energy efficiency.

## Abstract 2

Carbon footprints of metals have declined in high-income regions due to energy efficiency while increased in emerging economies due to economic growth.

## Introduction

### Climate Impact of Metals

**Figure 1:**
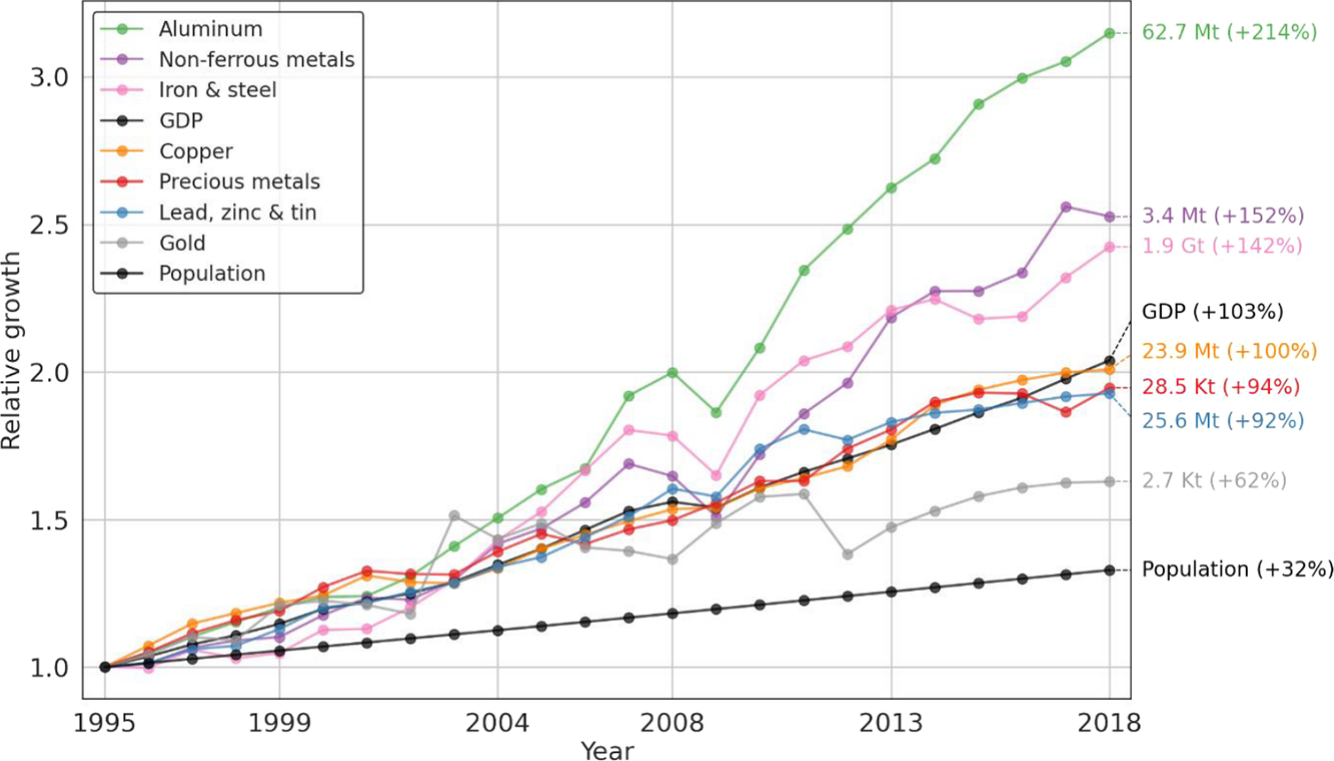
Global metal production, GDP, and population growth rates since 1995. GDP is in constant 2005 prices. Sources: British Geological Survey (metals production statistics), EXIOBASE (GDP), and UN WPP (population).

Materials, and especially metals, play a key role in the development of modern economies. While population only increased fourfold from 1900 to 2010,^1^ the rate of material extraction increased 11-fold.^2^ This discrepancy is mainly driven by a fivefold increase in per capita stock of manufactured capital, such as buildings, infrastructure, and durable goods.^2^ In the last couple of decades, production rates of all primary metals have increased significantly faster than global population (Figure 1). In the case of iron and steel, aluminum, and nonferrous metals, their rates have even grown substantially faster than the global gross domestic product (GDP). Especially aluminum stands out with a growth of 1.8 times the GDP growth. Gold is the only metal that has experienced stagnation for a prolonged period (more than 2 years) but has increased steadily in the last 10 years of this study.

Metal production has severe environmental impacts, both locally and globally. On a local scale, it is mainly a matter of waste and toxicity, while globally it has been recognized as a main contributor to climate change.^3^ The latter is due to both high process emissions and energy use in metal production and its supply chain. Direct process emissions are for a range of metals identified as sources of emissions that are particularly difficult to abate,^4^ while the high energy intensity of metal production uses roughly 8% of the total energy supply.^5^

Historically, greenhouse gas (GHG) emissions have been strongly coupled with increasing affluency through the increasing demand for goods.^6^ Some international organizations, governments, and scenario models argue or assume that decoupling between increasing affluency and GHG emissions is possible through the concept of green growth.^7−12^ On the other hand, some scientists have been critical of the possibility of a green growth pathway.^13−15^ It is however uncertain if such decoupling in a green growth scenario is universally feasible, or if it is only the case for specific metals and their GHG emissions.^16^ These emissions can be reduced through both technological developments and environmental policies.^12,17^ Since the early 1990s, efforts have been made in mitigating GHG emissions through international agreements.^18,19^ Studies have shown that emissions in the upstream supply chain of metals have increased significantly in the last two decades especially due to the rapid expansion of metal refining in China, which relies heavily on coal.^20−22^ However, it is not clear how different technological and socioeconomic drivers have impacted these emissions and what role they have played in a potential decoupling.

In the case of metals, two types of decoupling are of interest, namely, resource or impact decoupling. The former concerns with the reduction of the actual amount of metals used while increasing affluency (GDP per capita), while the later concerns itself with environmental impacts caused by metal use, either directly (process emissions, production waste, etc.) or indirectly (energy emissions, end-of-life waste, etc.).^23^ Both types of decoupling can be either absolute (affluency increases while the metal demand or GHG emissions decrease) or relative (affluency increases at a faster rate than the metal demand or GHG emissions). Decoupling studies can also further be categorized based on the economic scope (sectoral or economy-wide), timescale (short or long timescales), or geographical scope (limited or global).^24^ In a green growth scenario for metals, it is an absolute, global, economy-wide, and long timescale impact or resource decoupling that is necessary for sustainability. Otherwise, the problem will just be shifted spatially, to a different sector or region, or temporally, to the future.

The primary focus of this study is the technological and socioeconomic drivers of GHG emissions occurring in the supply chain of metals.

### Contribution of this Study

Decoupling between environmental impacts and economic activities has been studied intensely over the last few decades and recently several literature reviews have been compiled.^6,17,24,25^ In the case of metal use and its associated environmental impacts, the decoupling literature is however limited. These papers study (a) aggregate material footprints (i.e., no distinguishing between individual metals and other materials);^6,7,26−33^ (b) the impacts of the metal sector based on monetary flows instead of physical production values;^7,21,31^ (c) solely resource decoupling instead of impact decoupling;^7,26,27,29−35^ (d) domestic material consumption instead of material footprints;^7,29,30^ (e) metal ores instead of actual metal production in physical units;^20,21,28,30−32,35,36^ (f) only scope one and two emissions;^6^ (g) specific regions;^7,28,34^ or a combination of these. A subset of these papers also applies structural or index decomposition analysis (IDA), but these are all subject to point (a), (b), and/or (c)^6,27,28,31−33^ (see Supporting Information S1 for an overview of which limitations each study has). None of these papers study the drivers of climate change impact specifically associated with metal use on a regional and global level. This study fills that gap by further progressing on the findings of recent studies that have analyzed the supply chain impacts in material production and consumption.^20−22,36^ It does so in two ways. First, it provides a detailed IDA of emissions over time. Second, it explicitly uses metal production values in physical terms rather than relying on costs or extracted metal ores as an indicator. Thereby, it can more reliably identify a potential decoupling between economic activities and primary metal consumption.

In this study, the term metal consumption includes both the direct and indirect metal use. The latter includes, for example, metals used to produce machinery, which in turn is used to produce food that is consumed in an economy. Hence, the indicator is comparable to the apparent domestic consumption (ADC) indicator introduced by Bleischwitz et al.,^34^ rather than the traditional domestic material consumption indicator used in material flow analysis (MFA). The word metal footprint is avoided as this has historically been used to refer to metal ore footprints.^35^

## Materials and Methods

### Methods

#### Input–Output and Footprint Analysis

Input–output analysis has in the span of the last seven decades become one of the most powerful tools to account for environmental impacts along supply chains of products.^37^ It is especially the development of global multiregional input–output (MRIO) models that cover an extended period consistently, which has made it possible to not just account for impacts but also analyze their trends and drivers across increasingly global production networks.^38−41^

The footprint of a specific final demand is calculated using the Leontief demand model:
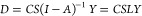
1where *Y* is the final demand matrix, *L* the Leontief inverse matrix, *S* the stressor/emissions intensity matrix, *C* is the characterization matrix, and *D* is the impacts/footprints.^42^ The data required to construct these matrices are from national statistics, trade databases, and agencies, which monitor either socioeconomic or environmental stressors. Eq 1 can be used to calculate any type of footprint for any final demand sector or aggregate. Additionally, the footprint can be attributed to different stages of the supply chain by modifying the equation.^36^

However, to calculate the scope three emissions of the products a certain sector in the economy produces, the standard demand model is not sufficient. Instead, the hypothetical extraction method (HEM) is required.

#### Upstream Energy Use and Emissions of Metals

The environmental footprints of metals cannot be determined with the demand-driven Leontief model that is used for common footprint analysis, because most metal use is as an intermediate product. To identify the influence of the activity level in one sector on that in another, Strassert^43^ proposed the HEM and Szyrmer et al.^44^ the total flow method (see also Sections 12.2.6 and 6.6.2 in Miller and Blair^45^). Gallego and Lenzen^46^ showed that the two methods are mathematically equivalent. Total flow, however, is quicker to calculate, while hypothetical extraction offers more information on the upstream supply chain. Cella^47^ further decomposed the HEM to identify the importance of the different linkages of the extracted sectors. Refining the work of Dente et al.,^48,49^ Cabernard et al.^20^ proposed the supply chain impact method to calculate the environmental footprints of materials. Hertwich^36^ showed that this method is mathematically equivalent to hypothetical extraction, and it can be shown to be a special case of eq 12 as provided by Cella.^47^ The supply chain method, however, has the advantage of being computationally faster to calculate and simpler to interpret.

In the following study, the terminology/nomenclature presented by Cabernard et al. is adopted.^20^ Matrices are subscripted with letters that indicate which rows or columns of the model are not removed. Subscript T refers to target sectors, O refers to nontarget sectors, and all refers to all sectors in the model. Hence, a matrix with subscript (T, O) will have only target sectors as rows and nontarget sectors in the columns. Equivalently, if no subscript is given, then it is assumed to be regular matrix of the IO model, i.e., (all, all).

Thus, the upstream impact of each of the target sectors can be calculated as
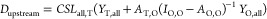
2

Furthermore, the results are of interest on an individual regional level, so the final demand matrix, *Y*_O, all_, is summed to a vector for each region, *y*_O, reg_. Then, to get the upstream impact of each target sector separately, the calculation needs to be done for each element of that vector separately, which is done using the diagonal operator, **diag**. The equation used to calculate the footprints of the target sectors triggered by the final demand from a region then becomes
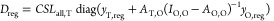
3

Then, using different *S* and corresponding *C* matrices for the different stressors of interest (primary metals, energy, and GHG emissions in this case) produces the values needed for the analysis. Eqs 2 and 3 are reformulations of eq 9 in Cabernard et al.^20^

Applying the method as described above provides for each region in the MRIO model, the amount of metal consumed both directly in final consumption and indirectly through the demand on other sectors, and the upstream impact of those metals. The results of these calculations are presented in the Supporting Information (S8).

#### Quantifying the Drivers

Once the upstream impacts of metals have been calculated, the drivers of the development of the consumption of the various commodities can be analyzed using decomposition analysis. Several IDA methods are available, and the reader is referred to de Boer and Rodrigues for an overview, derivation, and more detailed description of the methods.^50^ In this paper, the Montgomery (LMDI-I additive) method is used.^51,52^ IDA has been chosen over structural decomposition analysis (SDA) for two reasons. First, the study is an initial study of the upstream supply chain metal production emissions instead of the study of the structural changes of the economy. Second, an SDA would increase the dimensionality of the analysis and most likely introduce more noise to the results. The Montgomery method requires timeseries for a set of factors that are used to calculate an impact variable. As the interest of this paper is to understand the drivers of the climate impact of metal consumption, the following Kaya-like identity^53^ is decomposed
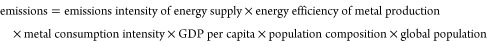
or mathematically
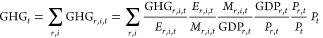
4where GHG is the GHG emissions in CO_2_ equivalents, *E* is the energy use, *M* is the primary metal (direct and indirect) use, GDP is the gross domestic product, and *P* is population. The index *r* indicates the region using the metal, *i* indicates the metal used, and *t* indicates the year (or year period). A missing index from the factors in the above implies that the value is aggregated over this index if applicable, i.e., *P_t_* is the global population (aggregated over all regions), but not aggregated across all years. A more detailed description of the Montgomery (LMDI-I additive) method is provided in the Supporting Information (S6) as well as a description on how to interpret the different drivers used in the decomposition (S7).

### Data Sources

Three data sources have been used to provide values for the analysis presented in this paper. The MRIO used is the product version of EXIOBASE 3.8.2.^54^ EXIOBASE has been chosen over other MRIOs as it has a higher resolution on metal products, based on industry-specific data sets. This means that the environmental impacts of each of these groups of metals can be analyzed separately. Furthermore, EXIOBASE provides multiple emissions and energy extensions, as well as GDP values for each region (in constant 2005 prices). The emissions and energy extensions used in this work are updated and yet to be released versions of the “GHG emissions AR5 (GWP100)|GWP100 (IPCC, 2010)” and “Energy Carrier Net Total” extensions, respectively. These newly updated extensions are based on updated IEA data instead of extrapolations. They are used in eq 3 above to calculate the values for GHG and *E* in eq 4.

Population data are taken from the United Nations World Population Prospects,^55^ which are then aggregated to the EXIOBASE region classification.

The World Mineral Statistics data set from the British Geological Survey (BGS) was used to construct an extension describing the production of primary metals in physical terms.^56^ An overview of all metals and their groupings can be found in the Supporting Information (S5). The data were downloaded, cleaned, structured, and matched to the mining and producing sectors in the MRIO model using the CPA2002 classification. The apparent consumption (i.e., use) of primary metals of each region and sector was calculated assuming homogeneous prices.

The analysis focuses on the 24 year period from 1995 to 2018. EXIOBASE, like any environmentally extended MRIO model, is based on a compilation of various sources and assumptions. Therefore, the modeling and balancing process does not always provide sensible signals when analyzing time series of ratios, where the nominator and denominators are based on data sets from different sources. Hence, to avoid highly fluctuating datapoints, due to the factors in the decomposition varying significantly across some years, the metal-level data (M, E, and GHG) has been summed, while the regional- and higher-level data (GDP and P) have been averaged over multiple year periods. Therefore, as an example, the metal consumption intensity is the period aggregate metal consumption per unit of period average GDP. The metal-level data is not aggregated in order to show the absolute increase in emissions between periods instead of yearly averages. The criterium for the period length is that it is a factor (divisor) of the total period investigated. To include as much of the period without having to deal with too highly fluctuating data, 6 year periods were chosen.

Data for all figures are provided in the Supporting Information (S9).

## Results

### Diverging Trends for High-Income and Lower-Income Countries

**Figure 2:**
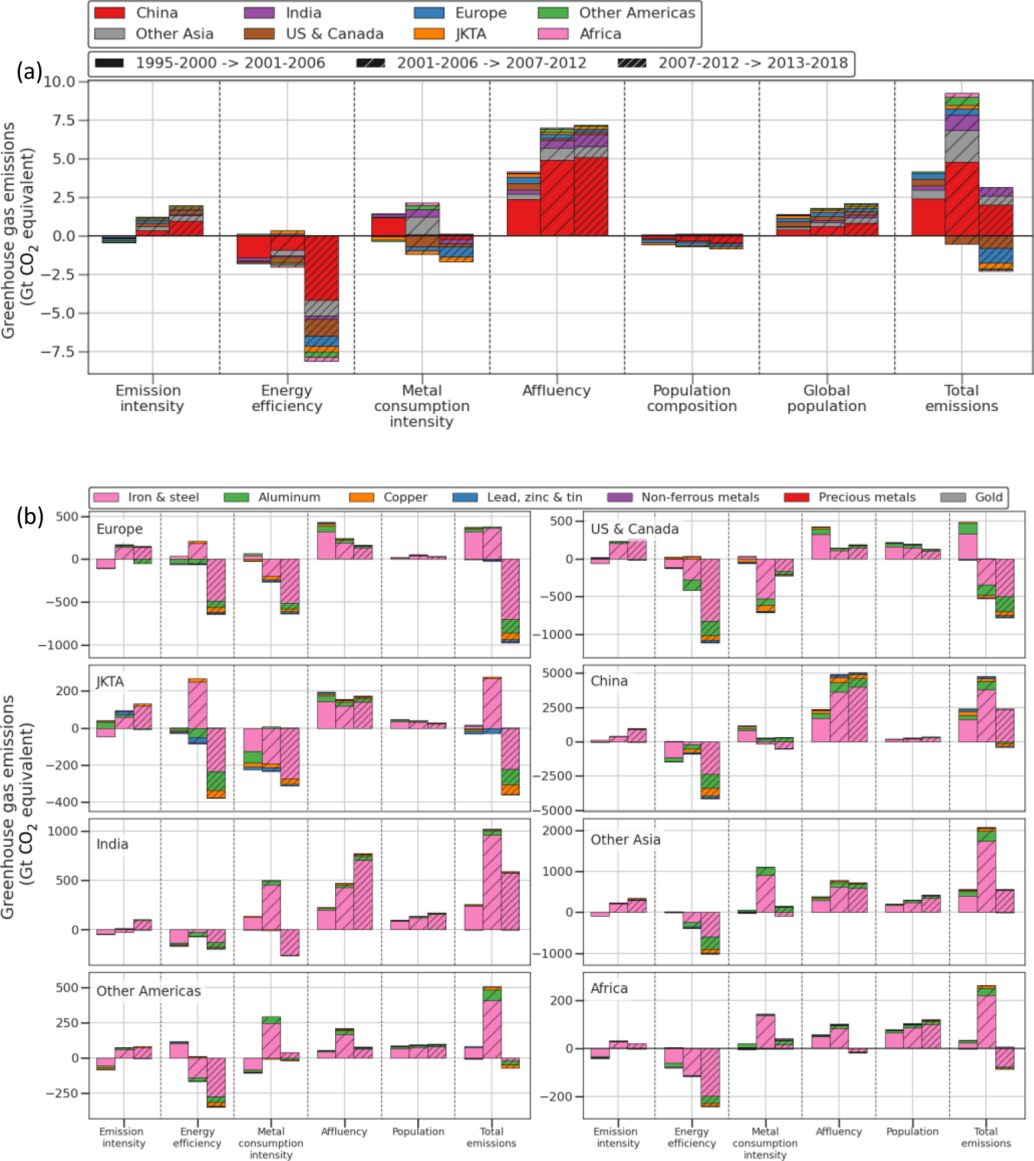
Montgomery (LMDI-I) decomposition of the GHG emissions in the upstream supply chain of metal production triggered by the consumption of metals in different regions. (a) Global decomposition with metal consuming regions in stacked bars. (b) Regional decomposition with metal groups in stacked bars. The factors in the decomposition are either 6 year aggregates (emissions, energy, and metals) or averages (GDP and population). Note: in (b), the subplots have different *y*-axis ranges.

On a global level, total emissions from metal production increased drastically in the first two periods (+4.17 and +8.73 Gt) but grew only slightly in the last period (+0.89 Gt) (Figure 2a). Global population and especially increasing affluency were driving up emissions in all the periods for all the regions. This is the case for both high-income and lower-income regions. Metal consumption intensity was a significant upward driver for lower-income regions in both the first period (+1.20 Gt for China) and the second period (+2.03 Gt for Africa, India, Other Asia, and Other Americas). Globally, the emission intensity changed from being a small downward driver in the first period (−0.36 Gt) to a notable upward driver in the last two periods (+1.21 and +1.97 Gt). Counterweighing these upward drivers improved energy efficiency (reduced energy per unit of metals) driving down emissions substantially for all regions, particularly during the last period (−8.10 Gt). In addition to this, metal consumption intensity in high-income regions (USA and Canada, Europe, JKTA (Japan, Korea, Taiwan, Australia)) during the last two periods drove down emissions (approximately −1.18 Gt in each period). The change in global population composition was only a minor upward driver.

The regional perspective provides a better insight into the trends for the specific metals consumed by each region (Figure 2b). The eight regions can, based on their trends, be grouped into three categories: (1) high-income regions (Europe, USA and Canada, and JKTA), (2) rapidly growing regions (China and India), and (3) other regions (Other Asia, Other Americas, and Africa).

The high-income regions are characterized by their total emissions decreasing when aggregating over all three periods. For Europe, the reduction mainly occurs in the last period (total changes across all periods −0.24 Gt), while the USA and Canada have net reductions in the last two periods (total changes across all periods −0.83 Gt), and JKTA has reductions in the first and last period (total changes across all periods −0.13 Gt). The primary cause for this reduction is reduced metal consumption intensity for almost all metals in all periods. Energy efficiency has been improving and has helped reduce emissions in the last period, but in the second period, it is a major upward driver for Europe (+0.19 Gt) and JKTA (+0.25 Gt) for iron and steel consumption. In the case of the USA and Canada, improved energy efficiency is the main reducing driver of emissions in the last period (−1.11 Gt). As on the global level, increasing affluency is the main driver of emissions for the USA and Canada and to some extent population growth. Iron and steel (pink bars in Figure 2b) dominate the emission changes for all regions, but aluminum (green bars) plays a relatively larger role for the high-income regions.

China was the dominant force behind the increase in upstream emissions and accounted for almost 66% of the global emission increase, whereas India accounted for 14% (third largest). China and India were distinct from the other groups by having increasing affluency as the key driver of emissions for all metals (total changes across all periods are +12.34 and +1.48 Gt for China and India, respectively). Change in metal consumption intensity is relatively unimportant for China but significant for India (+0.37 Gt). Improved energy efficiency has contributed to reduce the emissions for both China (total changes across all periods −6.47 Gt) and India (total changes across all periods −0.43 Gt).

The three other regions, Other Asia, Other Americas, and Africa, accounted for 23, 3.7, and 1.6%, respectively, of the absolute upstream supply chain emission increase (note: these numbers along with the shares of China and India sum up to more than 100% because the high-income regions have reductions in total emissions). These regions share trends but also each have their own traits. Metal consumption intensity, affluency, and population were all increasing drivers on roughly the same level in each region. Like for all other regions, improving energy efficiency was the main negative driver. While Other Asia had increasing total emissions in the last period, Other Americas and Africa had decreasing emissions. Africa distinguished itself from the other regions by having affluency as a negative driver in the last period. This is due to population outpacing economic growth in the region, leading to a reduction in per capita GDP.

A version of Figure 2a,b where the region and material dimensions have been swapped is provided in the Supporting Information (S2). It gives a better overview of the metal-specific trends on a global level.

### Peaking Metal Use in High-Income Regions

**Figure 3:**
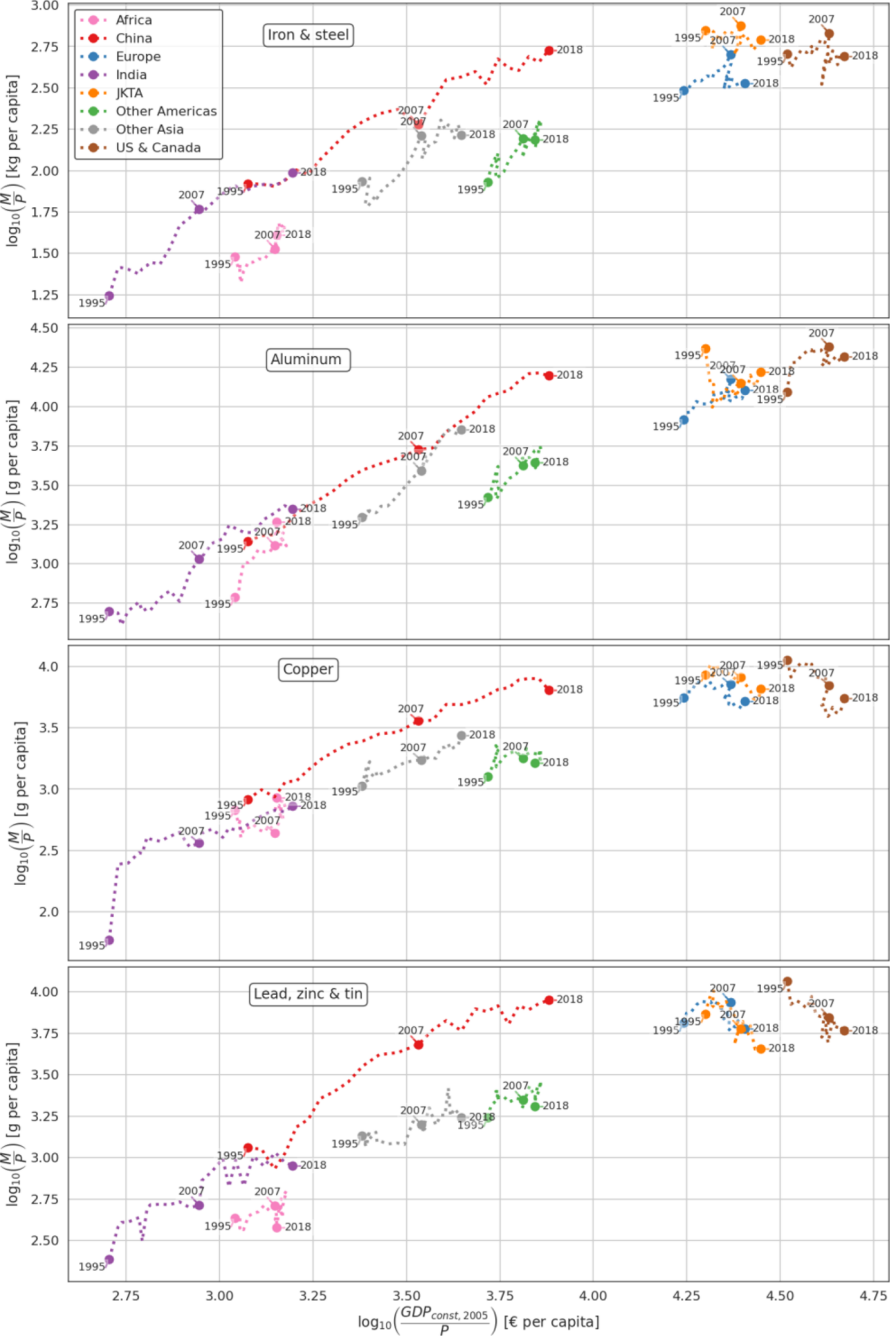
Development of metal consumption per capita rates plotted against GDP per capita for four metal groups.

The primary focus of the above analysis was GHG emissions. However, as one of the drivers, it traces primary metal consumption instigated by a region’s consumption through the bracketed part of eq 2. A declining metal consumption would provide for resource decoupling, a mechanism that has been emphasized by the International Resource Panel.^23^ In Figure 3 the trends for metal consumption and GDP (both in per capita) are compared for the different regions and for the most consumed metal groups (iron and steel, aluminum, copper, and lead, zinc and tin). Similar figures for the other metals are given in the Supporting Information (S4).

In accordance with previous literature, this study shows that high-income regions use significantly more metal per capita. Their consumption rates however were not notably higher in 2018 than in 1995. In the first half of the period, consumption of most metals increased gradually, before experiencing a large drop after the year 2007. Beyond this, the consumption rates only increased slightly to reach the 1995 levels at 6.08–6.25, 4.73–668, and 330–630 kg per capita for lead, zinc, and tin; copper; and iron and steel, respectively. Though an exception to this is the consumption of aluminum, which has almost reached the level of 2007 at 12.6–21.8 kg per capita. The GDP per capita of high-income regions has however grown nearly every year. JKTA has a higher metal consumption compared to Europe and the USA and Canada for the main construction metals, iron and steel and copper, apart from aluminum, of which the USA and Canada consumes the most.

At the start of the period, China’s consumption rates were below those of Other Asia and Other Americas. China’s metal consumption grew drastically and in 2018, reached or surpassed the rate of the high-income regions, even though its GDP per capita is lower. The general trend is the same for the consumption of all metal types apart from precious metals, which has not reached the same level as for the high-income regions. India, which also has experienced significant relative growth in metal consumption, has still moderate per capita consumption rates. In 1995, it had the lowest consumption rates of all regions for all metals but over the course of the study, only surpassed Africa. For most metals, India in 2018 barely surpassed the 1995 consumption rates of China and Other Asia. In 2018, the consumption rates were around 85.8, 1.58, 0.58, and 0.96 kg per capita for iron and steel, aluminum, copper and lead, and zinc and tin, respectively. The only outlier here is the consumption of precious metals, which almost reached the level of 2018 China and Other Asia at 2.48 g per capita. India’s slow growth in per capita levels might also be explained by its rapid population growth or low per capita income.

Africa has experienced growth in the first part of the period, but both metal consumption and GDP per capita have stagnated after 2007. Population growth in Africa has been significantly higher than for other regions and may therefore mask the absolute growth (in both metal consumption and GDP). Nevertheless, Africa has the lowest consumption rates for all metals, apart from copper and nonferrous metals, for which it is just slightly higher than the consumption rates of India. In 2018, consumption rates were 38.7, 1.34, 0.69, and 0.28 kg per capita for iron and steel, aluminum, copper and lead, and zinc and tin, respectively. The consumption rate of gold was an outlier for the case of Africa, for which it is higher than India, Other Asia, and Other Americas. This may in part be due to Africa having a large production of gold, but that the trade is partially untraced.

Both consumption rates and GDP per capita of Other Asia have grown substantially. For all metals but lead, zinc, and tin, their consumption rates have surpassed or reached the consumption rates of Other Americas, despite their GDP per capita being smaller. Consumption rates of Other Americas have fluctuated over the period of the study. The consumption rates dropped in the same years as GDP per capita stagnated. Hence, there appears to be strong coupling between the two. Three such stagnation periods occurred around the years 2000, 2008, and 2014 for Other Americas. In 2018, the consumption rates of Other Asia and Other Americas was around 146–147, 4.09–5.96, 1.53–1.87, 1.56–2.10 kg per capita for iron and steel, aluminum, copper and lead, and zinc and tin, respectively.

## Discussion

On a global level, there appears to be sparse evidence for absolute decoupling between economic growth, metal consumption, and the embodied impacts of the latter. The decomposition results of this paper clearly align with previous findings on increasing affluency being the main driver of environmental impacts.^6,17,25^ Population growth is an important driver of the emissions but also a driver that few governments try to influence directly. Historically, urbanization and increasing affluency in a region have been the most effective way of reducing population growth.^57^ However, there is an immediate cost in terms of resource use, as increasing affluency in turn drives up metal consumption to a higher per capita level. An interesting finding is that global population composition is a negative driver (Figure 2a). It means that the share of the global population living in regions whose carbon footprint of metal use is lower than average is increasing.

Metal consumption intensity is a decisive factor, which is also easier to influence through technological advancements or habitual changes.^11^ It was an emission-reducing driver in high-income regions. Contrarily, it was an emission-increasing driver for the lower-income regions except for China, where it had a small impact. The per capita values plotted in Figure 3 show how for the high-income regions, yearly metal consumption rates are stagnating or even decreasing while affluency is still increasing. These are however consumption rates; the stock of metals in the regions is still on the rise. While consumption rates appear to be increasing for aluminum, the rates seem to be slightly decreasing for iron and steel, copper, lead, zinc and tin, nonferrous metals, and gold. A recent MFA study that investigated both the ADC and stock for five of the largest economies (USA, UK, Germany, Japan, and China) in the period 1900–2013 found similar results of stagnation or reductions for steel, aluminum, and copper.^34^ The MFA study used the longer timeseries to determine saturation values for the different metals (kg per capita) at certain GDP/capita levels, which agree with metal consumptions presented in this study. In addition, the study suggests that ADC of China may surpass the saturation rates of the other economies temporarily, before saturating once the stocks are on the same level. This may explain why the metal consumption per capita of China has reached or surpassed the consumption rates of the high-income economies without having reached the same level of affluency, as found in this study. Furthermore, other MFA studies have also shown or predicted metal stocks to saturate once consumption rates reach a certain level.^58^ The future trajectory of China’s metal consumption is however uncertain. In the last few decades, China’s economy has been driven to a large degree by capital investment, which has resulted in a huge metal production capacity.^59^ This capacity is likely to become redundant as China plans to shift its economy to a more private consumption-based economy.^60^ The timing and speed of such a shift will have a substantial impact on China’s metal consumption trajectory and to what degree it will follow one of the high-income regions in the coming decades. A study has shown that use of a more varied spectra of metals is highly correlated with the GDP, Human Development Index, and Global Competitiveness Innovation index.^61^ This could be an explanation for why precious metal consumption levels of China are significantly lower than the high-income regions, despite having the same level of consumption for most other metals.

It may also be that a future recoupling occurs for most economies. A panel data analysis has shown that since 1970, dematerialization only occurred on a national level during economic recessions.^62^ These dematerialization periods have only meant a temporary slowdown in material consumption before growth resumed. However, the panel data analysis looked at aggregated material demands, and the case of metals may be different. In this study, it is apparent that the 2009 financial recession had an immense impact on metal consumption in high-income regions. The recession could also be the reason for the energy efficiency being such a pronounced upward driver for Europe and JKTA in the second period due to shifts in supply chains. However, this will remain as a question for future investigation as this study does not explicitly analyze supply chain effects in the decomposition analysis.

The decomposition results showed that it is mainly high-income regions, which have managed to reduce their emissions embodied in metal consumption. This is unsurprising for three reasons. First, these regions have the highest metal consumption rate per capita and thereby the largest potential for reduction. Second, they have also built most of their essential infrastructure in the recent century, whereas the other regions still require metals for such infrastructure to develop their economies and improve the well-being of their population.^63^ Other IO studies have shown that the global carbon footprint of metals is increasing due to rising infrastructure in especially China,^20,36^ where there is a strong reliance on coal in industry.^21^ Third, the high-income regions are the main driving forces behind the climate deals of the recent decades.^18,19^ These may bring changes in consumption patterns and services, which in turn may reduce metal consumption and the associated environmental impacts.^3,64−66^ However, consumption patterns are often bound to culture and may take long to change profoundly. An example of such cultural tradition that heavily impacts metal consumption is that houses become valueless after one generation in Japan.^67^ Hence, old houses are demolished, and new metal is needed to build a new house. This may be a reason for the relatively high metal consumption rate of JKTA when compared to Europe and the USA and Canada, where old houses are investments that usually increase in value. Risk of natural disasters may also play a larger role for the JKTA region in general, as these regions are in a part of the world with a high risk of earthquakes and cyclones. Hence, constructions need to be sturdier and will therefore require more metal.^68^

Improved energy efficiency has led to reduction in all regions and especially China. Significant economies of scales have been observed in the steel industry,^69^ so the global improved energy efficiency could partially be explained by China’s increasing share of global steel production and export.^56^ Furthermore, the rapid growth in China’s steel production meant a shift to the newest production technology. A study estimated that such a shift to the most energy efficient technologies could reduce the global energy demand for steel by 10%.^22^ The increasing concentration of global steel production in China would also explain why the emission intensity is an upward driver for all regions (Figure 2b). As previously mentioned, China’s metal industry relies more on coal energy^21^ and would therefore lead to increasing emissions, despite the shift to newer production technologies. In the future, emissions intensity may become a more impactful driver, as regions increase the share of renewable energy sources.^70,71^ However, such a green transition will likely increase the metal demand significantly, depending on the energy technologies that are invested in ref (72). Hence in short term, metal consumption intensity may increase and therefore also the associated emissions. Furthermore, the transition to renewable energy sources will only reduce energy-related emissions and not the direct process emissions. These must instead be addressed through advancements in the production technologies.^11^ In the case of iron- and steelmaking, various technologies are currently being developed and tested.^73^ Even if these new technologies become economically viable, it will likely be decades before current iron and steel plants become obsolescent, due to their long lifetimes.

A higher utilization of secondary metals instead of primary metals may also provide a fruitful path forward, which in turn also avoids the increasing issue of metal ore yield degradation.^74^ The production of steel in electric arc furnaces is an example of a secondary metal production technology that can be powered by renewable energy sources and therefore emits significantly less GHG emissions. It has been estimated that a shift to secondary steel and aluminum could reduce the emissions by 10–38 and 3.5–20%, respectively.^22^ However, the scope of this paper is only limited to primary metals due to data limitations.

In the high-income regions, there is evidence of relative resource decoupling between affluency and metal consumption and absolute impact decoupling between affluency and the emissions associated with metal production. This is predominantly due to reduced metal consumption intensity and improved energy efficiency. On the contrary, there is a strong coupling in lower-income regions, in spite of improved energy efficiency in metal production. Some studies suggest that these are likely to decouple once the regions reach similar levels of per capita metal stocks as the high-income regions. The evidence for decoupling presented in this study provides optimism for future green growth scenarios. However, for high-income regions, the impact decoupling is only evident on a short timescale, while the relative resource decoupling in high-income regions has stabilized at a high consumption level. If other regions are to reach the same level of metal consumption rates before decoupling, it may take decades before absolute decoupling occurs on a global level. A more rapid decline in emissions is required for metal production to be compatible with the Paris Agreement.

## Supporting Information Available

Grouping of metals (S5) (XLXS)
Detailed HEM analysis results (S8) (XLSX)
Data for all figures (S9) (XLSX)
Overview of related studies, extra figures and tables, HEM analysis results, LMDI-I index decomposition, interpretation of the drivers, and results of HEM analysis (PDF)
