# Supplementary material for: Decomposition Analysis of the Carbon Footprint of Primary Metals
Source: Environ Sci Technol. 2023 May 5;57(19):7391–400. doi: 10.1021/acs.est.2c05857 (PMC10193522; doi:10.1021/acs.est.2c05857)
Supplement: Supplementary file 4 — es2c05857_si_004.pdf [file es2c05857_si_004.pdf]

Decomposition Analysis of Carbon Footprint of Primary Metals

Supporting information

Kajwan Rasul*,

Edgar Hertwich

Industrial Ecology Programme, Department of Energy and Process Engineering, Norwegian University of Science and Technology, Høgskoleringen 1, Trondheim 7034, Norway

*Email: kajwan.rasul@ntnu.no

Contents

A decomposition analysis of the carbon footprint of primary metals 0
List of tables 0
List of figures 0
S1: Overview of limitations of related studies 1
S2: Decomposition analysis with region and metal dimensions swapped 2
S3: Regional decomposition analysis in per capita terms 3
S4: Metal consumption per capita vs GDP per capita for other metal groups 4
S5: Grouping of metals, regions, and sectors 5
S6: Montgomery (LMDI-I) additive index decomposition 5
S7: Interpretation of the drivers 6
S8: HEM analysis results 7
S9: Data used in figures 10
References 10

List of tables

Table S1: *Overview of limitations of related studies*
Table S2: *HEM analysis results*

List of figures

Figure S1: *Decomposition analysis with region and metal dimensions swapped*
Figure S2: *Regional decomposition analysis in per capita terms*
Figure S3: *Metal consumption per capita vs GDP per capita for other metal groups*

S1: Overview of limitations of related studies

Limitation	(a)	(b)	(c)	(d)	(e)	(f)	(g)	(h)
Bleischwitz et al. 2018[1]			X				X	X
Cabernard et al. 2019[2]					X			X
Cabernard et al. 2022[3]		X			X			X
Hellweg et al. 2019[4]								X
Hertwich 2021[5]					X			X
Krausmann et al. 2009[6]	X		X	X				X
Krausmann et al. 2018[7]	X		X	X	X			X
Lamb et al. 2021[8]	X					X		
OECD 2011[9]	X	X	X	X			X	X
Plank et al. 2018[10]	X		X					
Plank et al. 2021[11]	X				X		X	
Pothen et al. 2017[12]	X	X	X		X			
Schandl et al. 2018[13]	X		X		X			
Schandl et. al 2019[14]	X		X					
Wiedmann et al. 2015[15]	X		X					X
Wiedmann et al. 2015[16]			X		X			X

Table S1: Limitations are (a) aggregate material footprints (i.e., no distinguishing between individual metals and other materials); (b) the impacts of the metal sector based on monetary flows instead of physical production values; (c) solely resource decoupling instead of impact decoupling; (d) domestic material consumption instead of material footprint; (e) metal ores instead of actual metal production in physical units; (f) only scope 1 and scope 2 emissions; (g) specific regions; (h) no decomposition analysis.

S2: Decomposition analysis with region and metal dimensions swapped

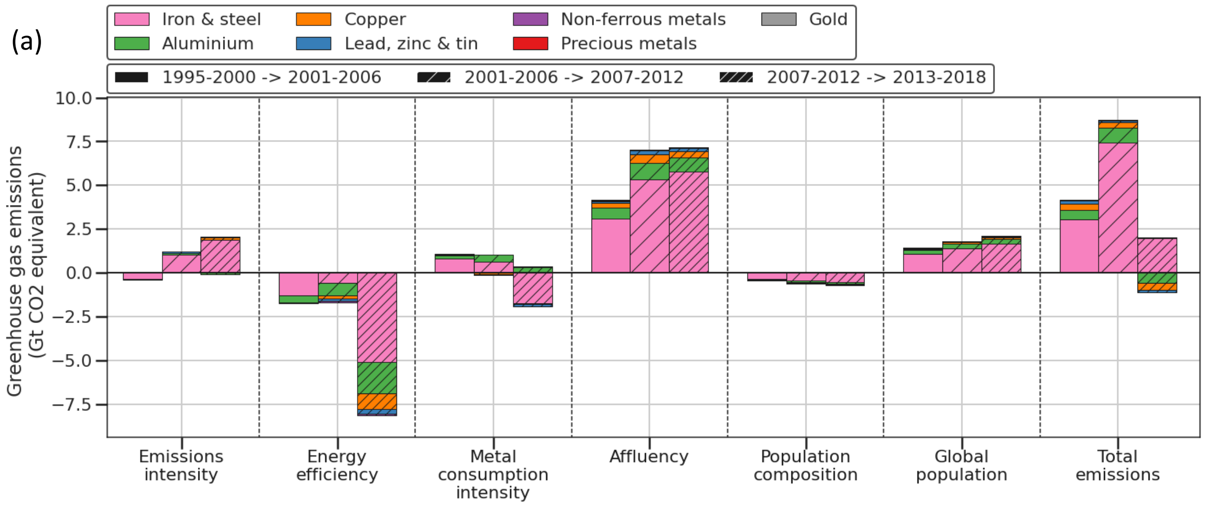

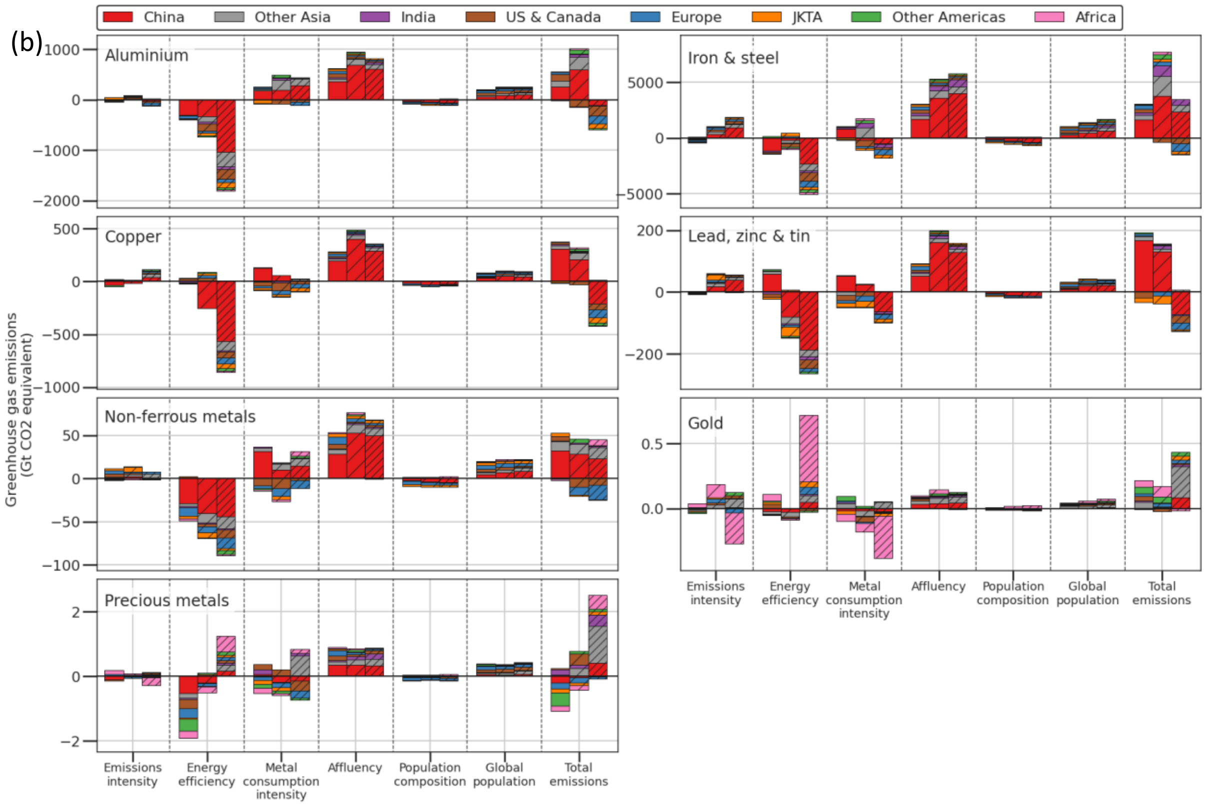

Figure S1: Montgomery (LMDI-I) decomposition of the GHG emissions in the upstream supply chain of metal production triggered by the consumption of metals in different regions. (a) Global decomposition with different metal groups in stacked bars. (b) Metal group level decomposition with regions in stacked bars. The factors in the decomposition are either 6-year aggregates (emissions, energy, and metals) or averages (GDP, population). Note: In (b) the subplots have different y-axis ranges.

S3: Regional decomposition analysis in per capita terms

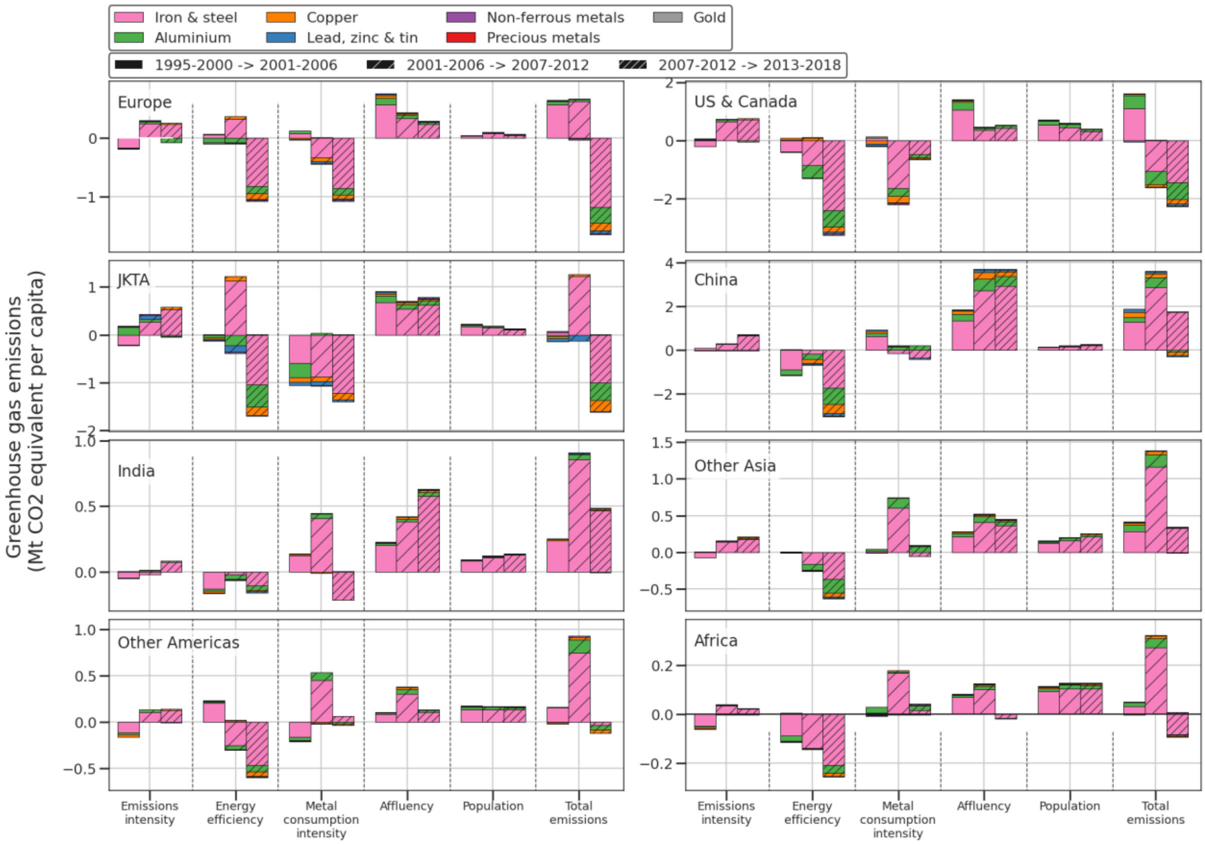

Figure S2: Montgomery (LMDI-I) decomposition of the GHG emissions in the upstream supply chain of metal production triggered by the consumption of metals in different regions. Metal groups are distinguished between in stacked bars. Note: the y-axis ranges differ for the different regions. The factors in the decomposition are either 6-year aggregates (emissions, energy, and metals) or averages (GDP, population). Note: In (b) the subplots have different y-axis ranges.

S4: Metal consumption per capita vs GDP per capita for other metal groups

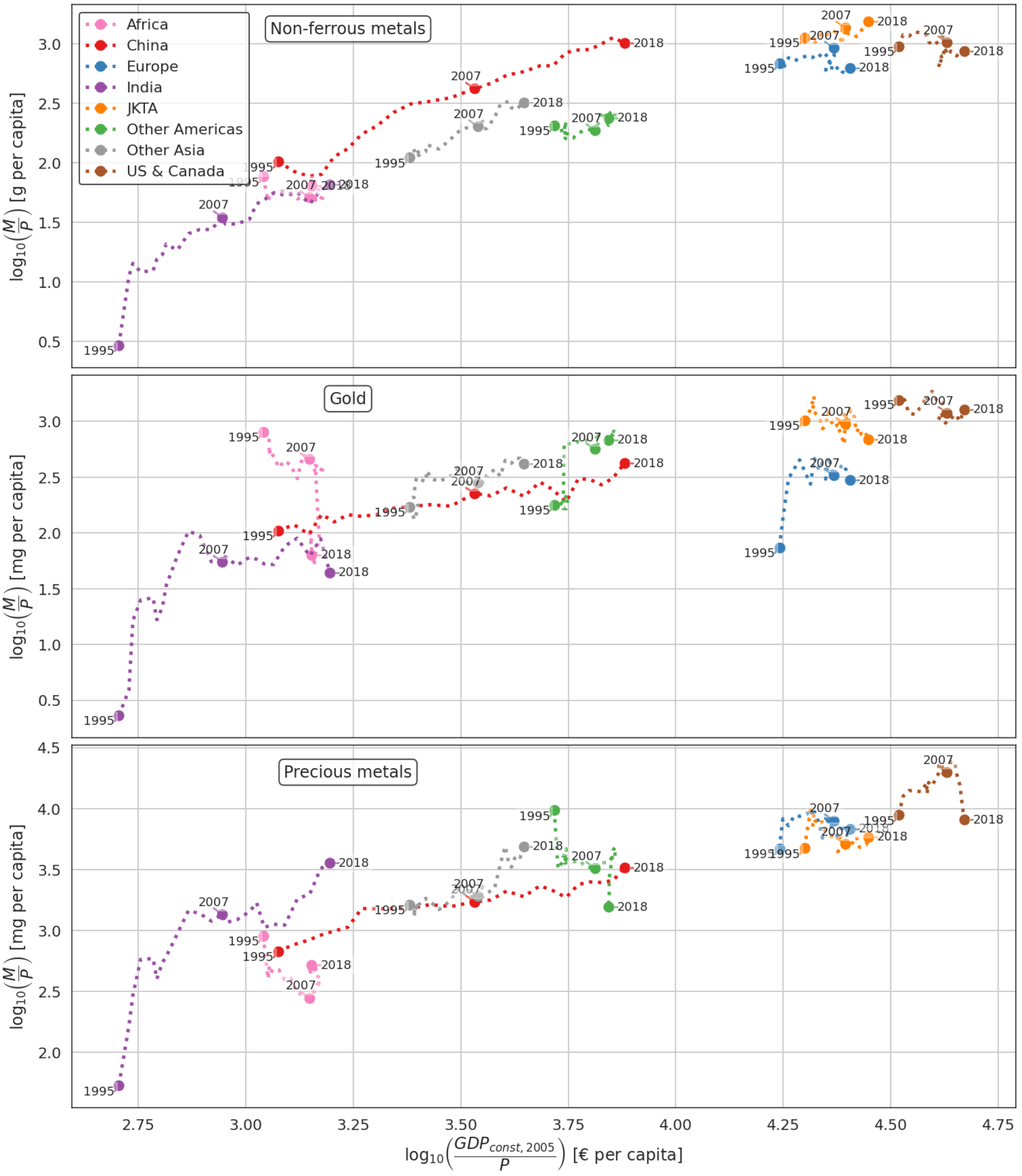

Figure S3: The development of metal consumption per capita rates plotted against GDP per capita for three metal groups not included in the main paper.

S5: Grouping of metals, regions, and sectors

See attached S5.xlsx file.

S6: Montgomery (LMDI-I) additive index decomposition

In the following, let each factor in Equation (4) be written as x_k, where subscript k runs over the set of factors, K, in the equation, e.g. $x_{k,r,i,t} = \left\{\frac{GHG_{r,i,t}}{E_{r,i,t}}, \frac{E_{r,i,t}}{M_{r,i,t}}, \dots\right\}$. Then the equation can be rewritten as

$$GHG_t = \sum_{r,i} GHG_{r,i,t} = \sum_{r,i} \prod_{k \in K} x_{k,r,i,t} \tag{5}$$

For the Montgomery (LMDI-I additive) method, the weights, w^M, are calculated as

$$w^M = L\left(GHG_{r,i,t+1}, GHG_{r,i,t}\right)$$

Where L is the logarithmic mean function defined as

$$L(a,b) = \frac{a-b}{\ln\left(\frac{a}{b}\right)}$$

The effect of each factor, y, is then given by

$$Y_{x_k}^M = \sum_{r,i} w^M \ln\left(\frac{x_{k,r,i,t+1}}{x_{k,r,i,t}}\right)$$

And the decomposition for the Montgomery (LMDI-I additive) method is then given by

$$D^M = \sum_{k \in K} Y_{x_k}^M$$

S7: Interpretation of the drivers

The emissions intensity is representative of how environmentally sustainable both the metal producing technology and the energy, used to produce it, is. A positive value indicates that there has been made a shift to less environmentally sustainable technologies or fuels. Contrary, negative values indicate a reduction in the carbon intensity of the fuel mix or an increase in the efficiency of the energy supply.

The energy efficiency reflects the efficiency of the technology used to produce the metals, including the energy needed to extract the metal from the ore. A negative value for the driver could be interpreted as technology improvements, whilst positive values could be due to decreasing metal ore grades or shift from more to less efficient production facilities. Though, the variations could also simply be due to a shift in the types of metals consumed.

The metal consumption intensity driver is a measure of how dependent an economy is on metals, both directly and indirectly. A negative trend here may be interpreted as relative decoupling of metal consumption and GDP, i.e., what is often discussed in the literature as a transition to a service-oriented economy. Additionally, it may be due to technological improvements in either the metal producing sector or the downstream sectors, e.g., less rebar is needed to produce a similar house by the construction sector due to improved rebar strength.

Affluency (GDP per capita) reflects the effect of wealth on metal consumption. The population composition shows the effect of population growing at varying rates across the globe.

S8: HEM analysis results

			Mean values			Shares	
			1995-2000	2013-2018	Growth (%)	1995-2000	2013-2018
Metal demand (Kg per capita)	First post-metal production product stage	Industry	95.2	155	+63	66.2	62.4
		Structures	34.8	71	+104	24.2	28.5
		Other	13.8	22.7	+64	9.6	9.1
		Total	144	249	+73		
	Final product stage	Industry	61.7	112	+81	45.2	46.7
		Structures	42.3	82.3	+94	31	34.4
		Other	32.3	45.2	+40	23.7	18.9
		Total	136	239	+75		
Energy use (TJ per capita)		Metal	1.71	2.9	+69.7	33.0	44.5
		Utilities	2.51	2.68	+6.7	48.4	41.1
		Other	0.97	0.94	+2.3	18.6	14.5
		Total	5.18	6.52	+25.8		
Emissions (kg $CO_{2,\ equivalent}$ per capita)		Metal	186	346	+86.1	40.4	51.0
		Utilities	191	243	+27.2	41.5	35.8
		Other	83.8	89.5	+6.8	18.2	13.2
		Total	461	679	+47.2		

Table S2: (Top) Global metal demand by downstream production stage and product sector group. Mean values (kg per capita) and shares for the first and last six-year period is given, as well as the growth rate between these two averages. (Bottom) Corresponding values as for the above but for upstream energy use (TJ per capita) and greenhouse gas emissions (kg $CO_{2,\ equivalent}$ per capita) associated with metal production by different sector groups.

The hypothetical extraction method and footprint analysis provide a more detailed picture of the trends driving the emissions and energy use in metal production. First, they provide information on which downstream economic activities are driving the demand for the metals (based on monetary flows). The demand can be attributed to either (a) first post-metal production economic activities, i.e., where does the metal go in the economy right after being produced or (b) final product stage economic activities, i.e., what are the consumer products needing metals in their supply chain. Second, it shows which upstream activities uses energy and emits GHG emissions. Both results are shown on a global level in Table S2 (a regional version can be found in S8.xlsx). In the table, 6-year per capita average values for the first and last 6-year period, namely 1995-2000 and 2013-2018 are shown. In the following, all results are discussed in per capita terms, unless explicitly otherwise

stated. There are two reasons for this. First, it makes it easier to compare regions. Second, it will mask the population effect on the decoupling, hence make it more transparent if decoupling is occurring. Most future scenario models work under the presumption that global population growth will stagnate within the next 100 years, thus the population effect is not necessarily of interest.

Additionally, the growth rate between these two periods is provided. The downstream economic activities are aggregated into *industry, structures*, and *other*, while the upstream activities are aggregated into *metal, utilities,* and *other.* The share of these in the two periods are shown in Table S2.

The metal demand has grown 116 % and 73 % in absolute and per capita terms, respectively. For the high-income regions, the metal demand per capita has either remained unchanged (*Europe*) or fallen (*JKTA* -11 %, *US & Canada* -4.8 %). All other regions have increased their demand per capita, most notably *China* (+432 %) and *India* (+248 %). In the first post-metal production stage (including direct metal use), it is especially an increase in demand from *structures* that is driving the increase (+104 %), while demand from *industry* (+63 %) and *others* (+64 %) have been more moderate. Hence the share of metal demand from *structures* is increasing, but is still significantly lower than for *industries*, 28.5 % and 62.4 % respectively in the last period. On a regional level, *US & Canada* and *Europe* have roughly even shares of demand coming from *other* and *structures*, while for all other regions demand from structures is 3-5 times larger in 2013-2018. *India* is on this matter a clear outlier, with an immense increase in demand in *structures* (+2300 %). In comparison the second largest growth was less than 4 times lower in demand from *other* in *China* (+532 %). The share of demand from *structures* went from 10.4 % to 71.9 % for *India*. However, considering *India'*s metal demand from *structures* was exceptionally low (2.61 tons per capita) compared to the *Other Asia* region (26 tons per capita), the large growth is not too surprising. Despite the large growth *India*'s demand from *structures*, it only reached 62.8 tons per capita, which is just a bit more than *Other Asia* with 55.7

tons per capita. In comparison, *China* had 149 tons per capita demand from structures, which is only surpassed by *JKTA* with 162 tons per capita in 2013-2018.

Attributing the demand to final product stage before consumption instead, the picture is slightly different. Demand from *structures* has still grown the most (+94 %), but still less than the other perspective. It is different for *industries*, which has more demand on metals growth in its final product stage (+81 %). The *other* sector has only seen moderate growth (+40 %). Hence on a global level, the share of metal demand coming from *structures* and *industry* is increasing, while *other*'s share is falling. *Industry* makes up the biggest share of the demand, ranging from 41.6 % to 55.7 %, for most regions, with especially the more affluent regions having the largest *industry* share. Outliers are *JKTA* and *India* who have their largest share of demand, 44.9 % and 69.1 % respectively, from structures. *US & Canada* and *Europe* have a relatively high share of demand, 25.7 % and 32.6 % respectively, induced by *other*, while *structures* make up less of the share. For all other regions, *structures* make up a larger share than *other*.

Energy use upstream from metal production has only grown 26 % when comparing the two periods. This is mainly due to an 70 % increase in the use of energy by metal producing sectors, while energy use in *utilities* only grew 6.7 %. Energy use by *other* sectors decreased 2.3 %. Thus, the share of energy used directly by *metal* increased by 11.5 % and is now the largest upstream source of energy. This shift of share trend from *utilities* to *metal* is occurring in all regions but varies significantly in size. The high-income regions and *Africa* decreased their energy embodied in metal production in per capita terms by 22.2 (*JKTA*), 15.7 (*Europe*), 45.2 % (*US & Canada*), and 9.8 *(Africa)*. All other regions have seen an increase in per capita terms, ranging from 17 % in *Africa* to 167 % in *China*.

Emissions in upstream activities have grown 47.2 %, significantly more than the energy use. In the *metal* sectors, the emissions have grown 86 %, which is significantly higher than the 70 % increase in energy use by the *metal* sectors. On the other hand, emissions from *utilities* only grew 27 % and *other* grew 6.8 %. Comparably, to the energy use, the shift in shares trend is similar for all regions.

The affluent regions and *Africa* have had a decrease in total upstream supply chain emissions due to metal consumption. While *JKTA, Europe*, and *Africa* have seen a decrease of around 10-12 %, *US & Canada* has had a staggering 37 % decrease. *China* emissions have risen 209 %, which is significantly more than their increase in energy use (+167 %). In contrast, the emissions in *India* and *Other Asia* grew 152 % and 93 %, which is a bit more than their increase in energy use, 151 % and 74 %.

S9: Data used in figures

See attached S9.xlsx file.

References

(1) Bleischwitz, R.; Nechifor, V.; Winning, M.; Huang, B.; Geng, Y. Extrapolation or Saturation – Revisiting Growth Patterns, Development Stages and Decoupling. *Global Environmental Change* **2018**, *48*, 86–96. https://doi.org/10.1016/j.gloenvcha.2017.11.008.

(2) Cabernard, L.; Pfister, S.; Hellweg, S. A New Method for Analyzing Sustainability Performance of Global Supply Chains and Its Application to Material Resources. *Science of The Total Environment* **2019**, *684*, 164–177. https://doi.org/10.1016/j.scitotenv.2019.04.434.

(3) Cabernard, L.; Pfister, S.; Hellweg, S. Improved Sustainability Assessment of the G20’s Supply Chains of Materials, Fuels, and Food. *Environ. Res. Lett.* **2022**, *17* (3), 034027. https://doi.org/10.1088/1748-9326/ac52c7.

(4) Hellweg, S.; Pfister, S.; Cabernard, L.; Droz-Georget, H.; Froemelt, A.; Haupt, M.; Mehr, J.; Oberschelp, C.; Piccoli, E.; Sonderegger, T.; Sudheshwar, A.; Walker, C.; Wang, Z. Environmental Impacts of Natural Resource Use. In *Global Resources Outlook 2019: Natural Resources for the Future We Want*; International Resource Panel, 2019; pp 64–97.

(5) Hertwich, E. G. Increased Carbon Footprint of Materials Production Driven by Rise in Investments. *Nat. Geosci.* **2021**, *14* (3), 151–155. https://doi.org/10.1038/s41561-021-00690-8.

(6) Krausmann, F.; Gingrich, S.; Eisenmenger, N.; Erb, K.-H.; Haberl, H.; Fischer-Kowalski, M. Growth in Global Materials Use, GDP and Population during the 20th Century. *Ecological Economics* **2009**, *68* (10), 2696–2705. https://doi.org/10.1016/j.ecolecon.2009.05.007.

(7) Krausmann, F.; Lauk, C.; Haas, W.; Wiedenhofer, D. From Resource Extraction to Outflows of Wastes and Emissions: The Socioeconomic Metabolism of the Global Economy, 1900–2015. *Global Environmental Change* **2018**, *52*, 131–140. https://doi.org/10.1016/j.gloenvcha.2018.07.003.

(8) Lamb, W. F.; Wiedmann, T.; Pongratz, J.; Andrew, R.; Crippa, M.; Olivier, J. G. J.; Wiedenhofer, D.; Mattioli, G.; Khourdajie, A. A.; House, J.; Pachauri, S.; Figueroa, M.; Saheb, Y.; Slade, R.; Hubacek, K.; Sun, L.; Ribeiro, S. K.; Khennas, S.; de la Rue du Can, S.; Chapungu, L.; Davis, S. J.; Bashmakov, I.; Dai, H.; Dhakal, S.; Tan, X.; Geng, Y.; Gu, B.; Minx, J. A Review of Trends and Drivers of Greenhouse Gas Emissions by Sector from 1990 to 2018. *Environ. Res. Lett.* **2021**, *16* (7), 073005. https://doi.org/10.1088/1748-9326/abee4e.

(9) OECD. *Resource Productivity in the G8 and the OECD*; OECD: Paris, 2011. https://www.oecd.org/env/waste/47944428.pdf (accessed 2022-02-26).

(10) Plank, B.; Eisenmenger, N.; Schaffartzik, A.; Wiedenhofer, D. International Trade Drives Global Resource Use: A Structural Decomposition Analysis of Raw Material Consumption from 1990–2010. *Environ. Sci. Technol.* **2018**, *52* (7), 4190–4198. https://doi.org/10.1021/acs.est.7b06133.

(11) Plank, B.; Eisenmenger, N.; Schaffartzik, A. Do Material Efficiency Improvements Backfire?: Insights from an Index Decomposition Analysis about the Link between CO_2 Emissions and

Material Use for Austria. *Journal of Industrial Ecology* **2021**, *25* (2), 511–522. https://doi.org/10.1111/jiec.13076.
(12) Pothen, F. A Structural Decomposition of Global Raw Material Consumption. *Ecological Economics* **2017**, *141*, 154–165. https://doi.org/10.1016/j.ecolecon.2017.05.032.
(13) Schandl, H.; Fischer-Kowalski, M.; West, J.; Giljum, S.; Dittrich, M.; Eisenmenger, N.; Geschke, A.; Lieber, M.; Wieland, H.; Schaffartzik, A.; Krausmann, F.; Gierlinger, S.; Hosking, K.; Lenzen, M.; Tanikawa, H.; Miatto, A.; Fishman, T. Global Material Flows and Resource Productivity: Forty Years of Evidence: Global Material Flows and Resource Productivity. *Journal of Industrial Ecology* **2018**, *22* (4), 827–838. https://doi.org/10.1111/jiec.12626.
(14) Schandl, H.; West, J.; Lutter, S.; Lieber, M.; Fischer-Kowalski, M.; Lenzen, M.; Geschke, A.; Miatto, A.; Tanikawa, H.; Bringezu, S.; Schaldach, R.; Flörke, M.; Schüngel, J.; Hüfner, R. Drivers, Pressures, and Natural Resource Use Trends. In *Global Resources Outlook 2019: Natural Resources for the Future We Want*; International Resource Panel, 2019; pp 38–63.
(15) Wiedmann, T. O.; Schandl, H.; Lenzen, M.; Moran, D.; Suh, S.; West, J.; Kanemoto, K. The Material Footprint of Nations. *Proc. Natl. Acad. Sci. U.S.A.* **2015**, *112* (20), 6271–6276. https://doi.org/10.1073/pnas.1220362110.
(16) Wiedmann, T. O.; Schandl, H.; Moran, D. The Footprint of Using Metals: New Metrics of Consumption and Productivity. *Environ Econ Policy Stud* **2015**, *17* (3), 369–388. https://doi.org/10.1007/s10018-014-0085-y.
